# SHOEBOX Modulates Root Meristem Size in Rice through Dose-Dependent Effects of Gibberellins on Cell Elongation and Proliferation

**DOI:** 10.1371/journal.pgen.1005464

**Published:** 2015-08-14

**Authors:** Jintao Li, Yu Zhao, Huangwei Chu, Likai Wang, Yanru Fu, Ping Liu, Narayana Upadhyaya, Chunli Chen, Tongmin Mou, Yuqi Feng, Prakash Kumar, Jian Xu

**Affiliations:** 1 National Key Laboratory of Crop Genetic Improvement, Huazhong Agricultural University, Wuhan, China; 2 Department of Biological Sciences and NUS Centre for BioImaging Sciences, National University of Singapore, Singapore; 3 Key Laboratory of Analytical Chemistry for Biology and Medicine (Ministry of Education), Department of Chemistry, Wuhan University, Wuhan, China; 4 Plant Industry, Commonwealth Scientific and Industrial Research Organization, Canberra, Australia; VIB University Gent, BELGIUM

## Abstract

Little is known about how the size of meristem cells is regulated and whether it participates in the control of meristem size in plants. Here, we report our findings on *shoebox* (*shb*), a mild gibberellin (GA) deficient rice mutant that has a short root meristem size. Quantitative analysis of cortical cell length and number indicates that *shb* has shorter, rather than fewer, cells in the root meristem until around the fifth day after sowing, from which the number of cortical cells is also reduced. These defects can be either corrected by exogenous application of bioactive GA or induced in wild-type roots by a dose-dependent inhibitory effect of paclobutrazol on GA biosynthesis, suggesting that GA deficiency is the primary cause of *shb* mutant phenotypes. *SHB* encodes an AP2/ERF transcription factor that directly activates transcription of the GA biosynthesis gene *KS1*. Thus, root meristem size in rice is modulated by SHB-mediated GA biosynthesis that regulates the elongation and proliferation of meristem cells in a developmental stage-specific manner.

## Author Summary

Little is known concerning the identity of regulatory components and signaling pathways that control the growth of meristem cells in plants. Here, we report that rice plants deficient in the *AP2/ERF* family gene *SHOEBOX* (*SHB*) exhibited a severe reduction in the root meristem size. These plants had shorter cells in the root meristems following germination and fewer cells from approximately 5 days after sowing, suggesting that *SHB* regulates root meristem cell size and number in a developmental stage-specific manner and that cell size participates in the control of root meristem size in rice. *SHB* is positively regulated by GA signaling and encodes a direct transcriptional activator of the GA biosynthesis gene *KS1*, indicating that GA modulates its own biosynthesis and consequently the elongation of meristem cells in rice roots via positive feedback regulation on the transcription of *SHB* and *KS1*. Consistently, application of exogenous GA restored the size of root meristem cells to normal in *shb* and paclobutrazol-treated wild-type plants.

## Introduction

The size of a plant, or part thereof, is determined by combined activity of cell proliferation and growth during development [1]. Cell proliferation in plants occurs mostly in specialized tissues known as meristems, where new cells are produced to ensure that plants continue to grow in height and width throughout their life. Prior to mitosis, cells in the meristem must double in size by undergoing a slow but steady expansion in the direction perpendicular to the previous division plane, which enables them to divide and keeps the size of their daughter cells constant [2,3]. A more pronounced growth (denoted as post-mitotic cell expansion), however, is commonly seen in differentiating cells that are displaced from the meristem. The extent of post-mitotic cell expansion is generally well correlated with the magnitude of organ growth [4].

Cell proliferation and growth in plants are influenced by genetic, hormonal, and environmental inputs. While little is known about the molecular mechanisms that regulate the size of meristem cells, numerous molecular players, including members of the AP2/ERF family of transcription factors, have been demonstrated to control either cell proliferation or post-mitotic cell expansion. For instance, the Arabidopsis AP2 transcription factor AINTEGUMENTA (ANT) promotes cell proliferation by maintaining the meristematic competence of cells [5]. *ANT* activity is activated by ARGOS (for auxin-regulated gene involved in organ size), a novel transcription factor acting downstream of auxin signaling [6]. In rice, several AP2/ERF genes including *OsEATB* (for ERF protein associated with tillering and branching [7], *SUBMERGENCE 1A* (*SUB1A*) [8], *SNORKEL1* (*SK1*) and *SK2* [9], were reported to have roles in regulating internode elongation, which is primarily post-mitotic expansion of differentiating cells displaced from the intercalary meristem near the node. *SK1* and *SK2* were suggested to trigger internode elongation via GA in response to rising water level [9]. By contrast, OsEATB was found to restrict GA responsiveness during the internode elongation process by down-regulating the expression of the GA biosynthetic gene Os*CPS2* [7]; whereas *SUB1A* limits GA responsiveness during prolonged submergence by augmenting accumulation of the DELLA family of GA signaling repressors SLENDER RICE 1 (SLR1) and SLR1 Like 1 (SLRL1), thus restricting underwater internode elongation and enhancing submergence survival [10].

GA plays an important role in the regulation of cell proliferation and growth during plant development [11–13]. It has been recently established that GA modulates both the rate of cell proliferation and the extent of post-mitotic cell expansion [3,14–16]. Inhibition of GA biosynthesis, either genetically in the GA biosynthesis mutant *ga1-3*, or by means of chemical treatment using paclobutrazol (PAC), an inhibitor of GA biosynthesis [17,18], reduces substantially the rate of cell proliferation in the Arabidopsis root meristem [3,14,15]. GA was proposed to promote root growth in Arabidopsis by increasing elongation (expansion along the root axis) of both dividing and post-mitotic endodermal cells, thereby indirectly controlling division and elongation of other types of root cells and the overall root meristem size [3]. However, how this process is regulated at the molecular level remains unclear.

Here we report the discovery of a novel GA-dependent size-control mechanism in the rice root meristem. We show that root meristem size in rice can be regulated by the extent of cell elongation in the root meristem. SHOEBOX (SHB), an AP2/ERF transcription factor, plays a key role in this mechanism. SHB directly binds to and activates transcription of the GA biosynthesis gene *KS1* in the root meristem, leading to the local production of GA that promotes elongation of meristem cells following germination, thus ensuring meristem growth and phenotypic plasticity during early stage of meristem development. At a later stage, SHB-dependent and KS1-mediated GA biosynthesis also participates in the modulation of cell proliferation in the root meristem, indicating a developmental stage-specific function of SHB.

## Results

### The *shb* Mutation Reduces the Length of Meristem Cells and Consequently the Size of the Root Meristem in Rice

**Fig 1:**
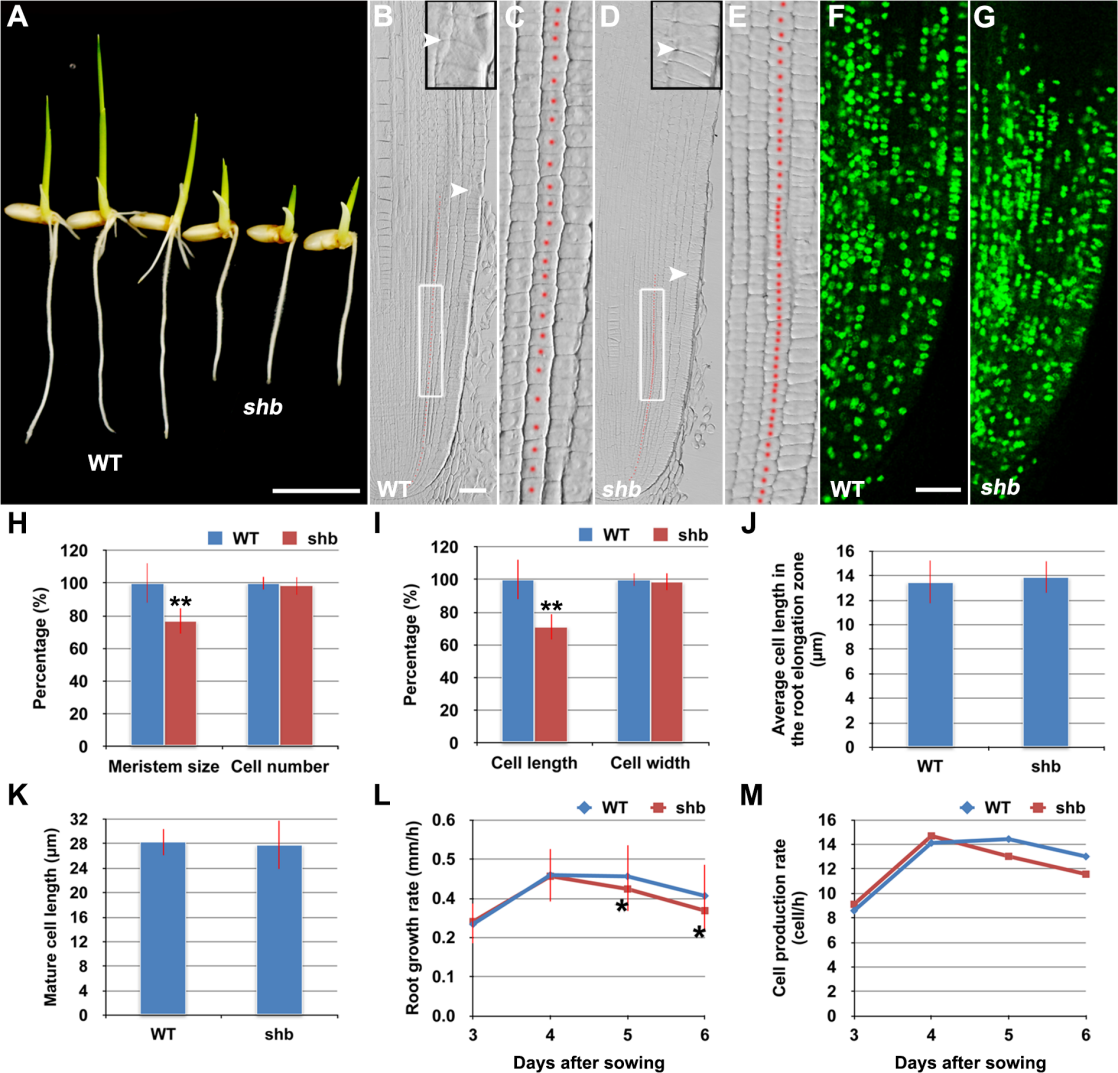
The *shb* mutation reduces the length of meristematic cortical cells and consequently the size of the root meristem in rice. (A) Phenotype of 4-day-old WT and *shb* seedlings. Scale bar = 1 cm. (B-E) Median longitudinal sections through root tips of 4-day-old WT (B, C) and *shb* (D, E) seedlings. Arrowheads indicate the proximal end of the root meristem. Insets are an enlargement of the regions at the proximal end of the root meristem. Red dotted line marks the 4^th^ cortical layer selected for the quantification analysis. (C, E) Boxed regions in (B, D). Scale bar = 50 μm. (F, G) Median longitudinal view of EdU staining in the root meristem of WT (F) and *shb* (G) seedlings. Scale bar = 50 μm. (H, I) Root meristem size (H), meristematic cortical cell number (H), average length (I) and width (I) of cortical cells in the root meristem of 4-day-old WT and *shb* seedlings. Data are expressed as percentage of the WT control, arbitrarily set to 100. Error bars represent SD (*n* = 15). **, *P* < 0.01, *t*-test. (J, K) Average length of cortical cells in the root elongation zone (J) and maturation zone (K). (L, M) Root growth rate and cell production rate in the root meristem of WT and *shb* seedlings. Measurement and calculation (cell production rate = root growth rate/ mature cell length) were performed at indicated days. Error bars represent SD (*n* = 15). *, *P* < 0.05, *t*-test.

In a rice enhancer trap screen we isolated a recessive mutant with a short primary root phenotype (Fig 1A), which we have named *shoebox* (*shb*; based on the shape of the cortical cells in the root meristem). Analysis of median longitudinal sections of root apices of 4-day-old wild-type (WT) and *shb* seedlings showed that the root meristem size of *shb* was shorter than that of the WT (Fig 1B and 1D and 1H). Quantification of cortical cell number and size in the root meristem of WT and *shb* mutant plants suggested that this was not due to a reduction in the number of meristematic cortical cells (Fig 1H), but was rather caused by a decrease in the length (but not width) of meristematic cortical cells (Fig 1C and 1E and 1I). Consistently, EdU staining indicated that the *shb* mutation did not noticeably alter cell proliferation in the root meristem (Fig 1F and 1G). Moreover, the average lengths of cortical cells in the root elongation and maturation zone did not differ between *shb* and the WT (Fig 1J and 1K), suggesting that *shb* has a root meristem-specific cell elongation defect. Notably, root growth rate and cell production rate in *shb* were not significantly altered in 3- and 4-day-old *shb* mutants but started to decline at around 5 days after sowing (Fig 1L and 1M).

### *shb* is a Novel GA-Deficient Mutant with a Mild Seed Germination Defect and Its Phenotypes Could Be Restored to WT by Exogenous Application of GA_3_

**Fig 2:**
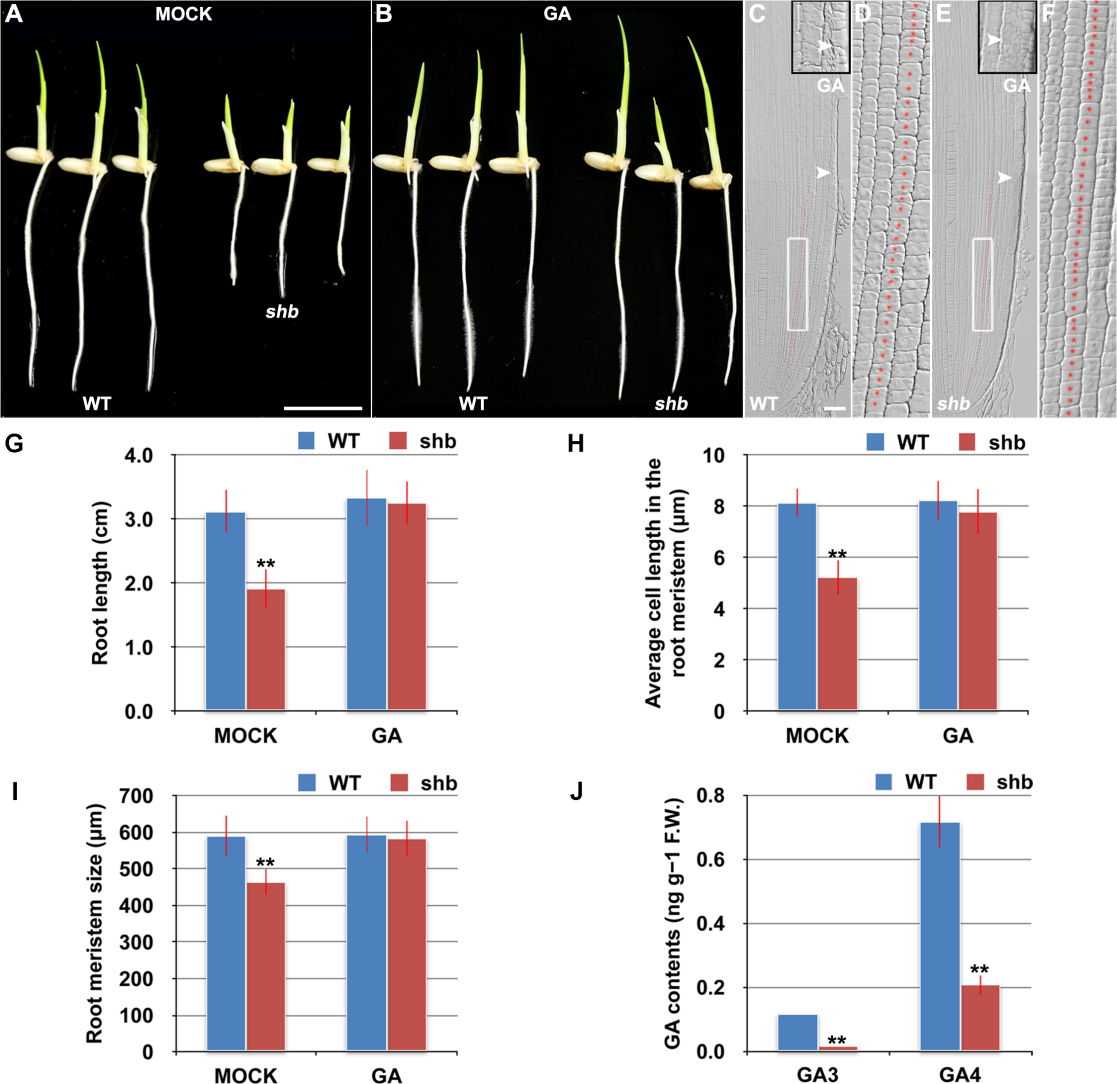
*shb* is a novel GA-deficient mutant whose root phenotypes could be restored to WT by exogenous application of GA_3_. (A) Phenotype of 4-day-old mock-treated WT and *shb* seedlings. Scale bar = 1 cm. (B) Phenotype of 4-day-old WT and *shb* seedlings grown on medium supplemented with 10 μM GA_3_. (C-F) Median longitudinal sections through root tips of 4-day-old GA-treated WT (C, D) and *shb* (E, F) seedlings. Arrowheads indicate the proximal end of the root meristem. Insets are an enlargement of the regions at the proximal end of the root meristem. Red dotted line marks the 4^th^ cortical layer selected for the quantification analysis. (D, F) Boxed regions in (C, E). Scale bar = 50 μm. (G) Root length of 4-day-old mock- and GA-treated WT and *shb* seedlings. Error bars represent SD (*n* = 15). **, *P* < 0.01, *t*-test. (H) Average cell length in the root meristem of 4-day-old mock- and GA-treated WT and *shb* seedlings. Error bars represent SD (*n* = 15). **, *P* < 0.01, *t*-test. (I) Root meristem size of 4-day-old mock- and GA-treated WT and *shb* seedlings. Error bars represent SD (*n* = 15). **, *P* < 0.01, *t*-test. (J) Endogenous levels of GA_3_ and GA_4_ in WT and *shb* roots. Error bars represent SD from three independent experiments. **, *P* < 0.01, *t*-test.

The aerial part of *shb* mutant plants has typical characteristics of rice GA-deficient or insensitive mutants [7,19,20], such as dwarfism and short internode length (S1 Fig). We thus hypothesized that the root phenotype of *shb* mutant plants might be caused by a defect in GA biosynthesis and/or signaling and examined whether it could be restored to WT by growing the mutants on medium supplemented with bioactive GA (GA_3_). 10 μM GA_3_ had no apparent effect on the WT control but could fully rescue the short-root phenotype of *shb* mutants (Fig 2A and 2B and 2G). Average length of cortical cells in the root meristem of *shb* was restored to that of the WT (Fig 2D and 2F and 2H), producing a root meristem with a similar size to that of the WT (Fig 2C, 2E and 2I). These results suggest that *shb* could properly respond to GA and GA deficiency is the primary cause of mutant phenotypes. In agreement with this suggestion, we found that the levels of GAs were reduced in *shb* roots as compared to the WT roots (Fig 2J). Particularly two bioactive GAs, GA_3_ and GA_4_, were significantly lower in *shb* compared to the WT controls (Fig 2J).

Because GA-deficient mutants often show delayed seed germination [21,22]. We next compared WT and *shb* seed germination and found that *shb* germinated approximately 12 h later than the WT (S2A–S2C Fig). As a result, 4-day-old *shb* had a markedly shorter root length compared to the WT controls when both seeds were sowed on medium at the same time (Figs 1A and 2A and 2G).

### *shb* Root Meristem Contains Shorter Cells following Germination and Fewer Cells from Approximately 5 Days after Synchronized Germination

To exclude the possibility that the short cortical cell phenotype observed in the *shb* root meristem was caused by delayed seed germination, we next synchronized WT and *shb* seed germination by sowing *shb* seeds on medium 12 h earlier before the WT control and performed a time-course analysis of various root phenotypes. We found that the sizes of root meristem (S2D Fig) and meristematic cortical cells (S2E Fig) were significantly and constantly shorter in *shb* than in the WT during the period of analysis. By contrast, meristematic cortical cell number (S2F Fig), root length (S2G Fig), root growth rate (S2H Fig) and cell production rate (S2I Fig) in the WT and *shb* were essentially identical until around the fifth day after synchronized germination, from which they started to diverge with significantly lower values in *shb* compared with the WT. Together, these results confirm our earlier observation that *shb* root meristem contained shorter cortical cells in the root meristem and suggest that fewer cells were produced from around the fifth day after synchronized germination. These phenotypes were not accompanied by changes in the lengths of elongation and maturation zone cells (S2J and S2K Fig), further demonstrating a specific role of *SHB* in the root meristem.

### *SHB* is Expressed in the Root Meristem and Encodes an AP2/ERF Transcription Factor

**Fig 3:**
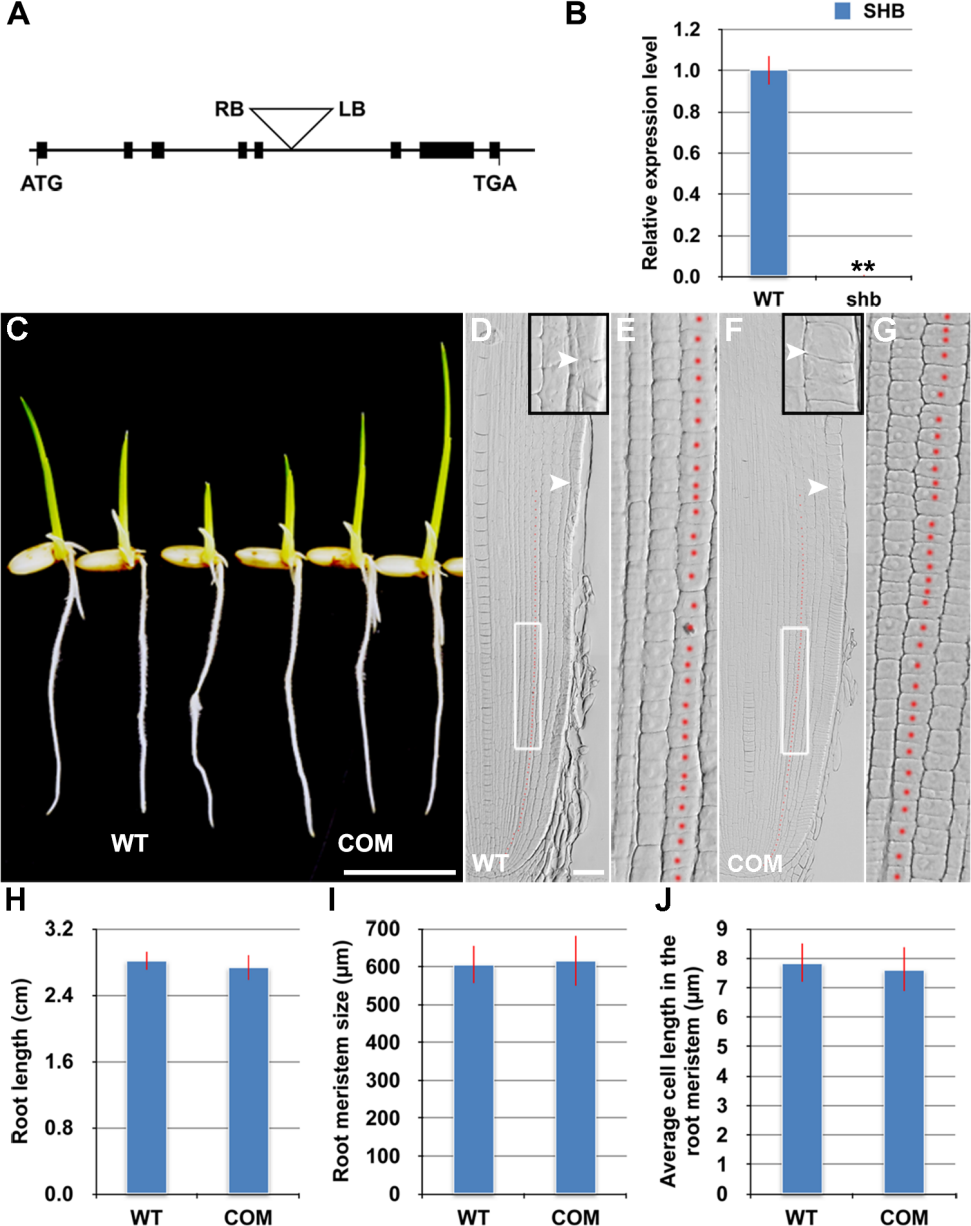
The root phenotypes of *shb* could be fully complemented by the *LOC_os05g32270* gene. (A) Schematic diagram of exon/intron structure of the *SHB* (*LOC_Os05g32270*) gene and T-DNA insertion site. Black boxes are exons. Introns are the open areas between the boxes. LB and RB represent the left and right borders of T-DNA. (B) qPCR analysis of transcript levels of *SHB* in roots of WT and *shb* mutant. Transcript levels from the WT were set to 1. Error bars represent SD from three independent experiments. **, *P* < 0.01, *t*-test. (C) Phenotype of 4-day-old seedlings of WT and a representative complementation line (COM). Scale bar = 1 cm. (D to G) Median longitudinal sections through root tips of 4-day-old WT (D, E) and COM (F, G) seedlings. Arrowheads indicate the proximal end of the root meristem. Insets are an enlargement of the regions at the proximal end of the root meristem. Red dotted line marks the 4^th^ cortical layer selected for the quantification analysis. (E, G) Boxed regions in (D, F). Scale bar = 50 μm. (H) Root length of 4-day-old WT and COM seedlings. (I) Root meristem size of 4-day-old WT and COM seedlings. (J) Average cell length in the root meristem of 4-day-old WT and COM seedlings. Error bars in (H) to (J) represent SD (*n* = 15). **, *P* < 0.01, *t*-test.

The *shb* mutant carries a homozygous T-DNA insertion in the 5^th^ intron of the *LOC_Os05g32270* gene (Fig 3A), which dramatically reduces the expression level of *LOC_Os05g32270* (Fig 3B). A genomic fragment containing the *LOC_Os05g32270* gene and its promoter and 3’ UTR regions could fully complement the mutant phenotypes of *shb* (Fig 3C–3J). We thus concluded that *LOC_Os05g32270* is the *SHB* gene.

The *SHB* gene has been previously termed *OsAP2-EREBP-049*, which encodes a putative transcription factor containing one AP2/EREBP DNA binding domain [23]. Based on the sequence similarity of the AP2/EREBP DNA binding domain, *SHB* was classified into the Group-1a of the AP2/ERF family, although all the other genes in this group have double AP2-EREBP DNA binding domains. A BLASTP search revealed that putative orthologs of SHB are present in both monocots and dicot plants (S3A Fig). The subsequent phylogenetic analysis suggested that SHB is more closely related to its putative orthologs in monocots than to its dicot counterparts (S3B Fig). Mutations in *DWARF & IRREGULAR LEAF* (*DIL1*), a homologous gene of *SHB* in maize, were reported to affect internode length, leaf shape and possibly root length [24], implying that *SHB* and *DIL1* have evolutionarily conserved functions.

**Fig 4:**
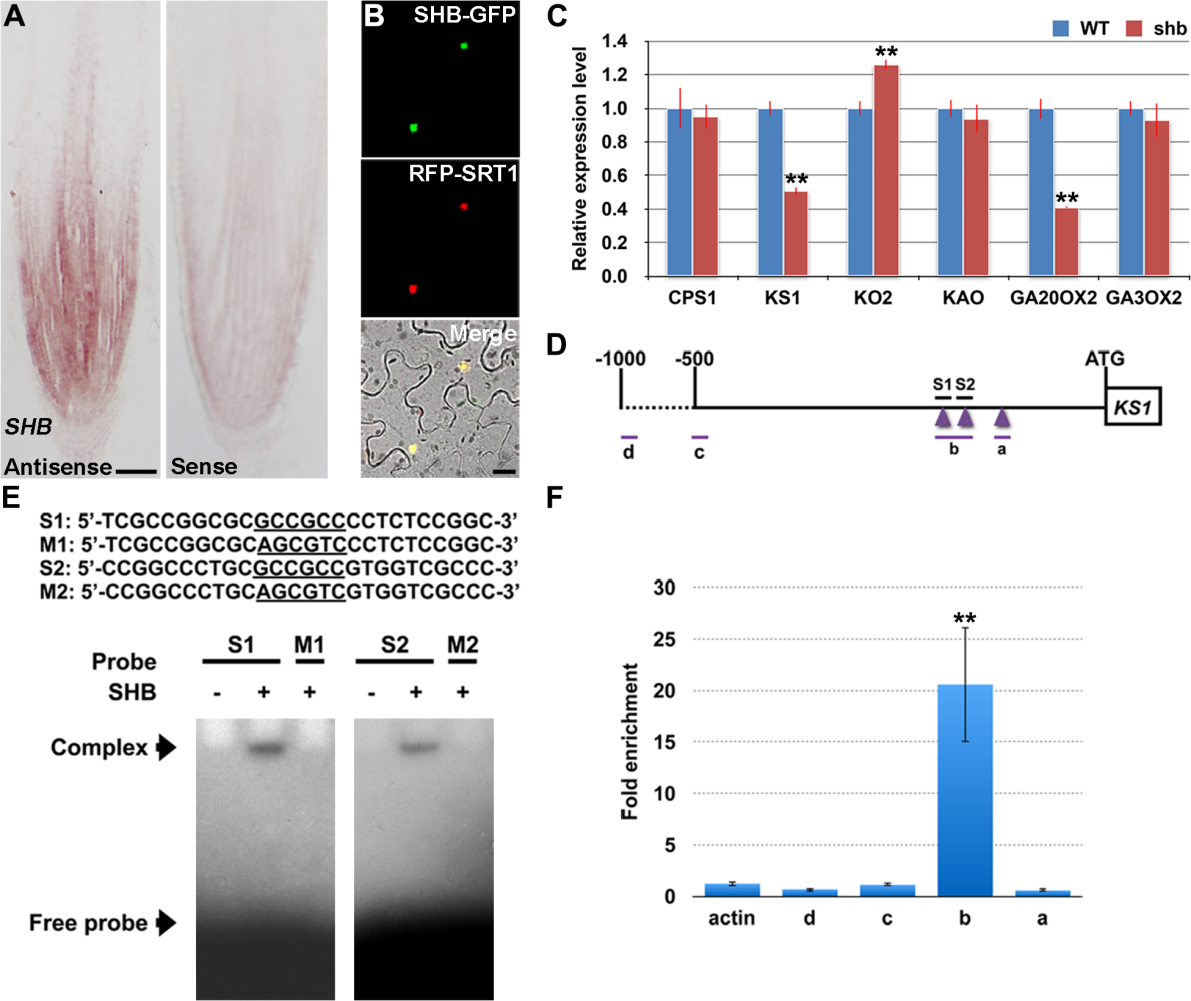
*SHB* is expressed in the root tip and encodes a nuclear-localized transcription factor that directly binds to and activates transcription of *KS1*. (A) RNA *in situ* hybridizations with an anti-sense probe (left panel) and a sense probe (right panel) showing *SHB* mRNA accumulation in the root tip. Scale bar = 100 μm. (B) Co-localization (lower panel; yellow) of SHB-GFP (upper panel; green) with a nuclear marker SRT1-RFP (middle panel; red) in tobacco epidermal cells. Scale bar = 20 μm. (C) qPCR analysis of transcript levels of GA biosynthetic genes *CPS1*, *KS1*, *KO2*, *KAO*, *GA20OX2*/*SD1* and *GA3OX2* in rice roots. Transcript levels from the WT were set to 1. Error bars represent SD from three independent experiments. **, *P* < 0.01, *t*-test. (D) Schematic diagram of *SHB* promoter region. Regions selected for EMSA and ChIP-qPCR experiments are shown by short lines and marked with letters. Purple arrowheads point to the position of GCC boxes. GCCGCC motif and the corresponding mutated DNA sequences are underlined. (E) Sequences of oligonucleotides used for EMSA, which indicates the binding of SHB to S1 and S2, but not to M1 and M2. (F) ChIP-qPCR analysis showing high level of association of SHB with fragment b. Error bars represent SD from three independent experiments. **, *P* < 0.01, *t*-test.

RNA *in situ* hybridization showed that *SHB* is expressed in the root meristem (Fig 4A), thus supporting a functional role for *SHB in vivo*. A fusion protein of SHB and GFP {SHB-GFP; under the control of the Cowpea Mosaic Virus (CPMV) promoter [25]} co-localized with a nuclear marker SRT1-RFP [26] in tobacco epidermal cells (Fig 4B), indicating that SHB functions as a nuclear-localized transcription factor.

### SHB Is a Direct Transcriptional Activator of the GA Biosynthesis Gene *KS1*

To determine the cause of GA deficiency in *shb*, we next examined whether the *shb* mutation decreases transcription of rice GA biosynthetic genes, including *CPS1*, *KS1*, *KO2*, *KAO*, *GA20OX2*/*SD1* and *GA3OX2*, by quantitative real-time PCR (qPCR). Among these genes, only *KS1* and *GA20OX2*/*SD1* were found to be significantly down-regulated by the *shb* mutation (Fig 4C), suggesting that SHB modulates the levels of bioactive GAs in rice roots through transcriptional activation of GA biosynthetic genes *KS1* and *GA20OX2*/*SD1*. Notably, the expression of *KO2* was weakly up-regulated in *shb*, perhaps to compensate for reduction of *KS1*, which is involved in an earlier step of the GA biosynthesis pathway.

AP2/EREBP proteins are able to bind the GCC-box, which is a short cis-acting element containing a core GCCGCC sequence motif [27]. Analysis of the *KS1* promoter identified three GCCGCC motifs located at 205, 184, and 131 nucleotides upstream to the translation start site (ATG; Fig 4D), whereas no GCC-box was found in the promoter region of *GA20OX2*/*SD1*. Electrophoretic mobility shift assay (EMSA) indicated that SHB could bind to GCCGCC motifs located at 205 and 184 nucleotides upstream to the ATG (S1 and S2; Fig 4D and 4E). No binding was detected with the third GCCGCC motif and SHB was not no longer able to bind to S1 and S2 when the two GCCGCC motifs were mutated (M1 and M2; Fig 4E).

To confirm the EMSA result *in vivo*, we performed ChIP-qPCR experiments with transgenic rice plants expressing a functional SHB-GFP fusion protein(S4 Fig). qPCR showed that fragment b, which contains GCCGCC motifs located at 205 and 184 nucleotides upstream to the ATG of *KS1*, was greatly enriched by ChIP with an anti-GFP antibody (Fig 4F). On the contrary, DNA fragments covering other regions of the *KS1* promoter, as well as the negative controls, were less amplified (Fig 4F). These data indicate that SHB can directly bind to two closely located GCCGCC motifs in the promoter region of *KS1 in vivo*.

### KS1-Mediated Local GA Biosynthesis Is Required for Dose-Dependent Effects of GA on Cell Elongation and Proliferation in the Root Meristem

**Fig 5:**
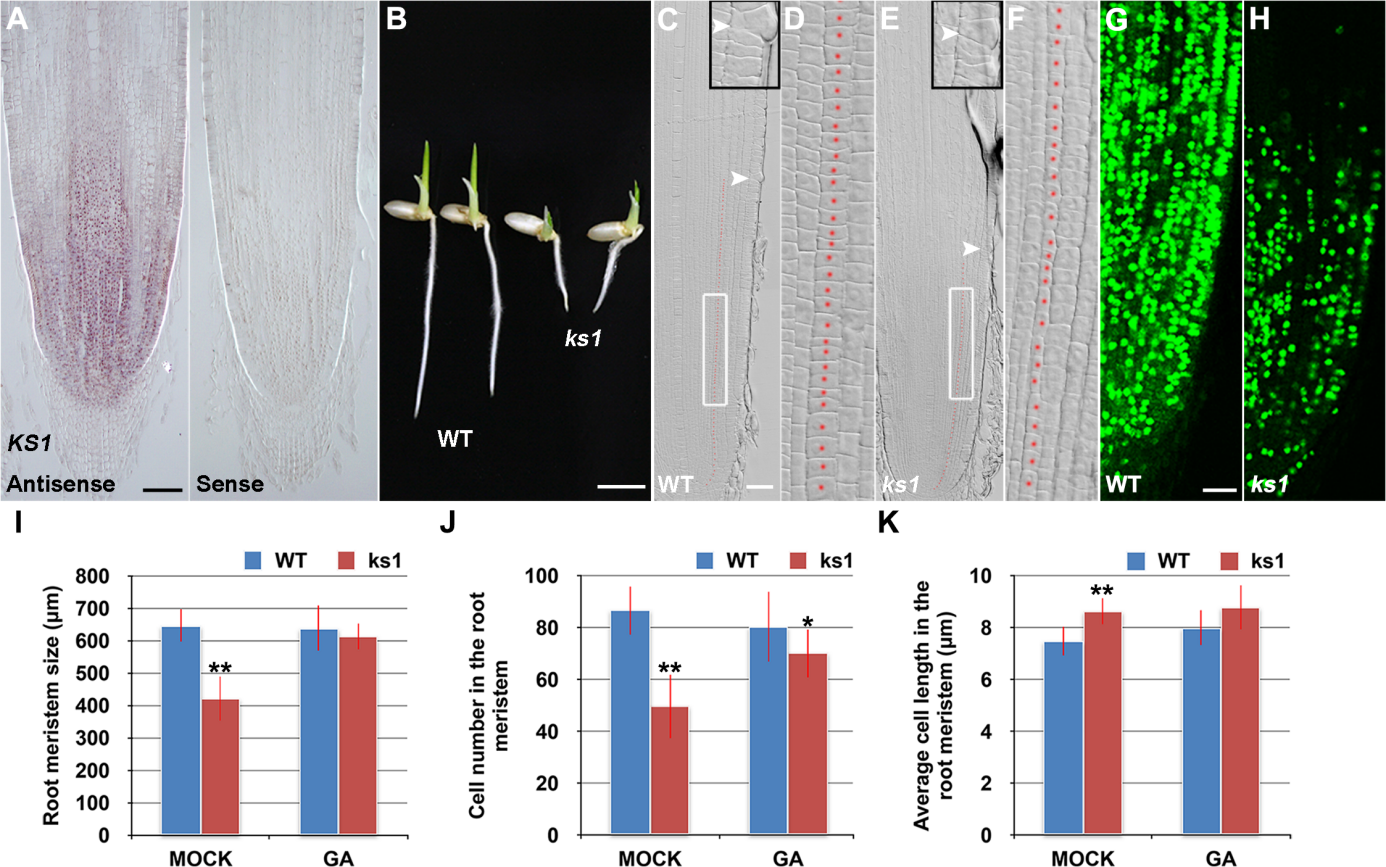
*KS1* is expressed in the root tip and has a role in GA-mediated elongation and proliferation of root meristem cells. (A) RNA *in situ* hybridizations with an anti-sense probe (left panel) and a sense probe (right panel) showing *KS1* mRNA accumulation in the root tip. Scale bar = 100 μm. (B) Phenotype of 4-day-old WT and *ks1* seedlings. Scale bar = 5 mm. (C-F) Median longitudinal sections through root tips of 4-day-old WT (C, D) and *ks1* (E, F) seedlings. Arrowheads indicate the proximal end of the root meristem. Insets are an enlargement of the regions at the proximal end of the root meristem. Red dotted line marks the 4^th^ cortical layer selected for the quantification analysis. (D, F) Boxed regions in (C, E). Scale bar = 50 μm. (G-H) Median longitudinal view of EdU-labelled cells in the root meristem of WT (G) and *ks1* (H) seedlings. Scale bar = 50 μm. (I) Root meristem size of 4-day-old WT and *ks1* seedlings treated with mock or 10 μM GA_3_. (J) Cell number in the root meristem of 4-day-old WT and *ks1* seedlings treated with mock or 10 μM GA_3_. (K) Average cell length in the root meristem of 4-day-old WT and *ks1* seedlings treated with mock or 10 μM GA_3_. Error bars in (I) to (K) represent SD (*n* = 15). **, *P* < 0.01, *t*-test; *, *P* < 0.05, *t*-test.

RNA *in situ* hybridization revealed that *KS1* was expressed in an overlapping domain with *SHB* (Fig 5A) and that *KS1* had reduced expression level and domain in the *shb* mutant (S5 Fig), in agreement with our finding that *KS1 is a* direct downstream target of *SHB*. A null *ks1* mutant with severe GA deficiency [19] was found to have shorter root length and smaller root meristem than the WT control (Fig 5B, 5C, 5E and 5I). 10 μM GA_3_ could fully complement the meristem size phenotype of *ks1* (Fig 5I), suggesting that KS1-dependent GA biosynthesis is essential for root meristem size control. Quantification of cortical cell number and size in the *ks1* root meristem revealed that there was a significant decrease in cell proliferation compared to the WT (Fig 5J), which was accompanied by an increase in the average length of cortical cells (Fig 5K), indicating cell cycle arrest [28]. EdU staining further confirmed the cell proliferation defect (Fig 5G and 5H) and 10 μM GA_3_ could largely restore the number of cortical cells in the *ks1* root meristem (Fig 5J), suggesting that severe GA deficiency in *ks1* is the underlying cause. Consistently, levels of most GAs were significantly reduced or undetectable in *ks1* seedlings compared to the WT and *shb* seedlings (S1 Table). Thus, we conclude that: 1) KS1-mediated GA biosynthesis is required for dose-dependent effects of GA on cell elongation and proliferation in the root meristem; and 2) The different root meristem phenotypes observed in *shb* and *ks1* mutants result from moderate versus severe GA reduction. The latter conclusion was also suggested by much greater up-regulation of *KO2* in *ks1* (S6 Fig) than in *shb* (Fig 4C) and in agreement with this conclusion, a higher concentration of GA_3_ was needed to restore in 24 hours the root meristem size in *ks1* (100 μM; S7A Fig) than in *shb* (50 μM; S7B Fig).

**Fig 6:**
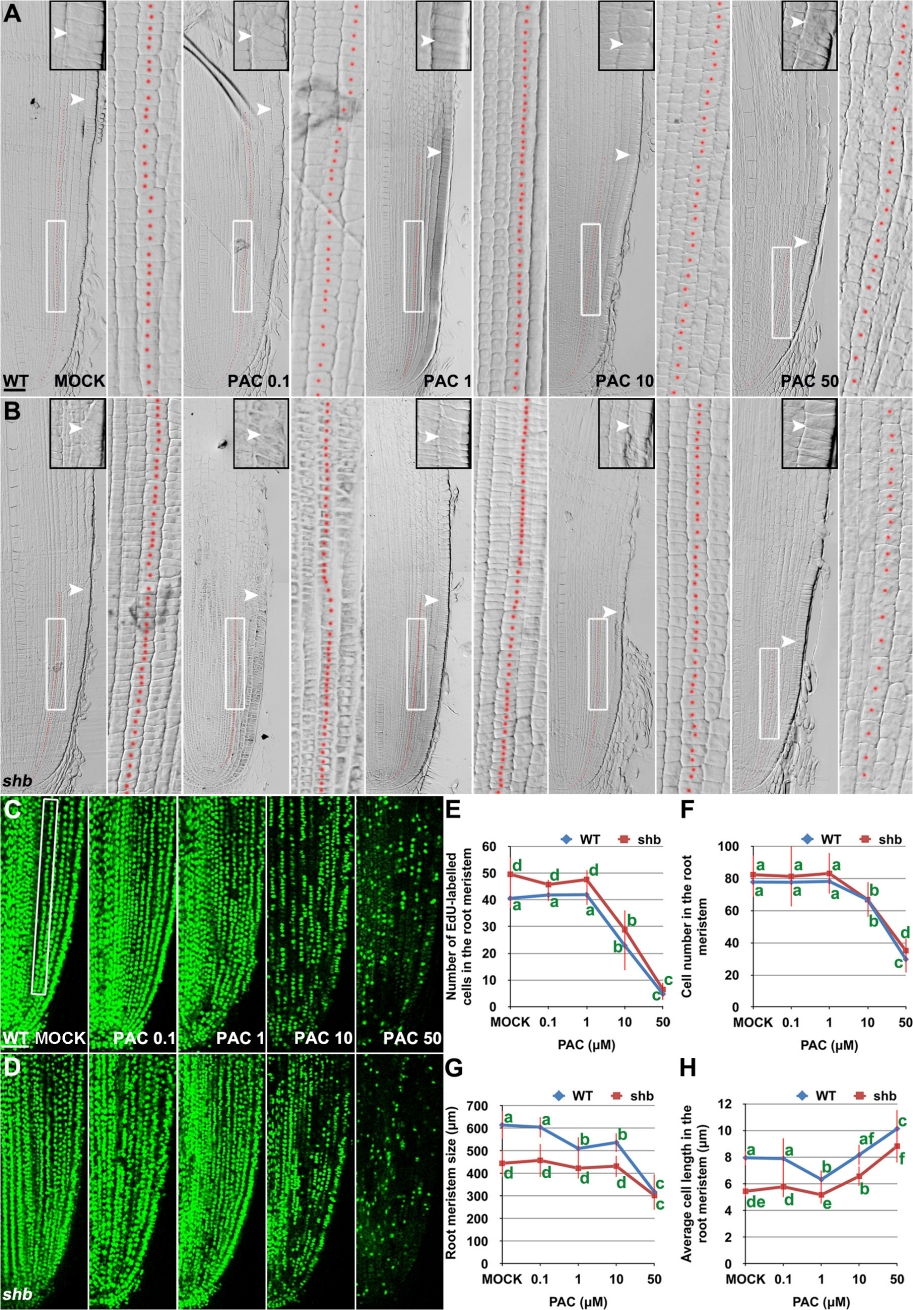
PAC has a dose-dependent effect on cell elongation and proliferation in the root meristem. (A, B) Median longitudinal sections through root tips of 3-day-old WT (A) and *shb* (B) seedlings treated with mock or indicated concentration of PAC for 24 hours. Arrowheads indicate the proximal end of the root meristem. Insets are an enlargement of the regions at the proximal end of the root meristem. Red dotted line marks the 4^th^ cortical layer selected for the quantification analysis. Scale bar = 50 μm. Boxed regions are magnified to show the size of root meristem cells in the 4^th^ cortical layer. (C, D) EdU-labelled cells in the root meristem of 4-day-old WT (C) and *shb* (D) seedlings treated with mock or indicated concentrations of PAC for 24 hours. Scale bar = 50 μm. (E) Number of EdU-labelled cells in the root meristem of 4-day-old WT and *shb* seedlings treated with mock or different concentrations of PAC for 24 hours. Quantification was performed in the 4^th^ cortical layer, in a selected portion (Boxed region in C) with a length of 360 μm. (F) Total cell number in the root meristem of 3-day-old WT and *shb* seedlings treated with mock or different concentrations of PAC for 24 hours. (G) Root meristem size of 3-day-old WT and *shb* seedlings treated with mock or different concentrations of PAC for 24 hours. (H) Average cell length in the root meristem of 3-day-old WT and *shb* seedlings treated with mock or different concentrations of PAC for 24 hours. Error bars in (E to H) represent SD (*n* = 10 for E and *n* = 15 for F to H). Bars with different letters are significantly different at *P* < 0.05, *t*-test.

Our results suggest that cell proliferation in the rice root meristem is regulated by a dose-dependent effect of GA. Consistently, severe inhibition of GA biosynthesis by 10 or 50 μM PAC significantly impaired cell proliferation in the root meristem of *shb* mutants and WT plants (Fig 6A–6F), resulting in a smaller root meristem (Fig 6A, 6B and 6G) with longer cells (Fig 6A and 6B and 6H). By contrast, 0.1 μM PAC had no obvious effects on *shb* mutants and WT plants (Fig 6A–6H), suggesting that PAC has a dose-dependent effect on cell proliferation. PAC-induced phenotypes could be reversed by co-treatment with GA (S8 Fig), confirming that they were caused by inhibition of GA biosynthesis by PAC. Notably, 1 μM PAC markedly reduced elongation of cortical cells in the WT root meristem (Fig 6H) without significantly affecting cell proliferation (Fig 6A–6F), whereas the root phenotypes of *shb* were less affected at the same concentration (Fig 6H). These observations together suggest that GA has a dose-dependent effect on cell elongation in the root meristem, which is regulated by SHB.

### SHB Functions as a Positive Regulator of GA Signaling

**Fig 7:**
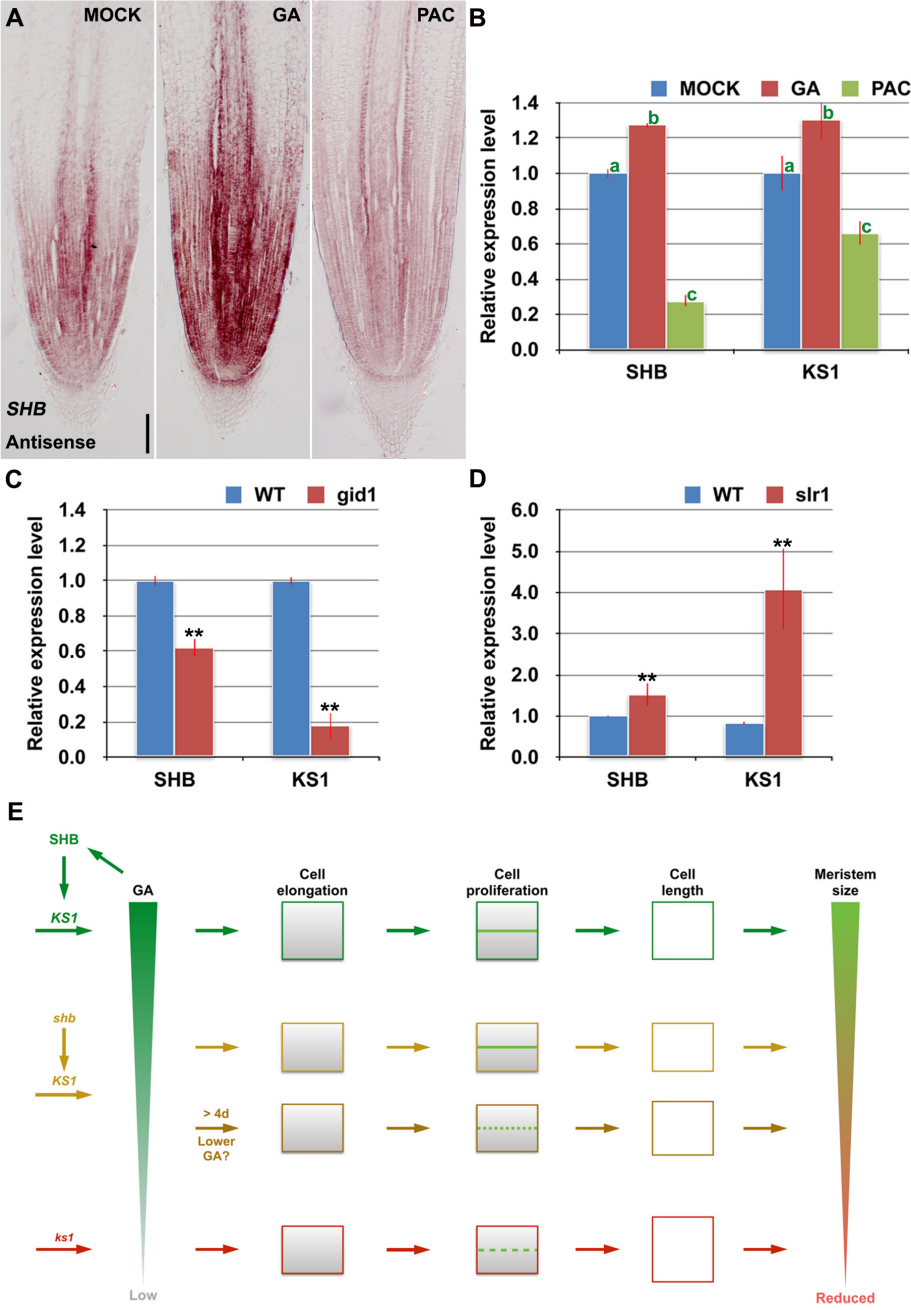
*SHB* functions a positive regulator of GA signaling and meristem growth. (A) RNA *in situ* hybridizations with an anti-sense probe showing *SHB* transcription was induced by GA and repressed by PAC in the root tip. Scale bar = 100 μm. (B) qPCR analysis of transcript levels of *SHB* and *KS1* in WT rice roots treated with mock, 10 μM GA_3_ or 10 μM PAC. Transcript levels from the mock were set to 1. Error bars represent SD from three independent experiments. Bars with different letters are significantly different at *P* < 0.01, *t*-test. (C, D) qPCR analysis of transcript levels of *SHB* and *KS1* in WT, *gid1* and *slr1* mutant roots. Transcript levels from the mock were set to 1. Error bars represent SD from three independent experiments. **, *P* < 0.01, *t*-test. (E) A model for meristem size control in the rice root. Root meristem size in rice is regulated through both SHB-dependent and independent GA biosynthesis pathways. SHB, whose transcription is induced by GA, promotes GA biosynthesis in the root meristem by directly binding to and activating *KS1*. SHB-dependent and KS1-mediated GA availability is well correlated with the ability of meristematic cortical cells to elongate beyond the minimal cell length requirement for normal cell proliferation, allowing optimal rice root meristem growth. Loss of SHB function reduces *ks1* transcription by half and consequently KS1-dependent endogenous GA levels, resulting in a shorter root meristem with apparent defect in cell elongation but not in cell proliferation at the early stage of meristem development. From around the 5^th^ day after sowing, the root meristem size of *shb* further decreases with fewer but longer meristematic cortex cells, indicative of cell cycle arrest. Loss of *KS1* function, which strongly reduces endogenous levels of GAs, results in a further reduced root meristem size due to a more severe reduction of cortical cell number and a further elongation of cortical cells. Thus, GA controls the root meristem size in rice through its dose-dependent effects on meristematic cell elongation and proliferation.

RNA *in situ* hybridization and qPCR showed that *SHB* transcription was induced by GA_3_ and repressed by PAC (Fig 7A and 7B), suggesting that *SHB* functions as a positive regulator of GA signaling. Consistently, the level of *KS1* transcripts was positively correlated with the level of GA (Fig 7B). Moreover, *SHB* and *KS1* expression was found to be down-regulated in the GA receptor mutant *gid1* [29] (Fig 7C), but up-regulated in *slr1* (Fig 7D), a constitutive GA response mutant defective in the *SLR1* gene, the only *DELLA* gene in rice [30]. Together, these results suggest that GA regulates its own biosynthesis via positive feedback regulation on the expression of *SHB* and *KS1*.

## Discussion

In this study, we have identified SHB, an AP2/ERF transcription factor, as a novel regulator of root meristem size in rice. We provide conclusive evidence that, during early stage of meristem development, root meristem size in rice can be regulated through SHB-dependent cell elongation, without the necessity to alter the rate of cell proliferation (Figs 1 and S2). This indicates that meristems, like organs, are able to adjust their size independent of cell number. A similar example can be found during *Drosophila melanogaster* imaginal disc development, during which the S6 kinase (dS6K) regulates cell size in a cell-autonomous manner without impinging on cell number [31]. From around 5 days after sowing, at which cell number in the root meristem, root growth rate and cell production rate start to decline (Figs 1I, 1J, S2F, S2H and S2I), however, SHB appears to regulate both cell elongation and cell proliferation in the root meristem.

SHB is required for longitudinal cell elongation but not radial cell expansion in the cortical layers of the rice root meristem (Fig 1I). Moreover, loss of *SHB* function did not affect the elongation of post-mitotic cells in the rice root (Figs 1J and S2J), indicating that *SHB* has a root meristem-specific function and that elongation of meristem cells differs in mechanism from rapid elongation of post-mitotic cells. This idea is in agreement with a previous report on the Arabidopsis *STUNTED PLANT 1* (*STP1*) gene, which is required for elongation of post-mitotic cells but not elongation of meristem cells [2]. *STP1* was found to mediate the effect of cytokinin on the elongation of post-mitotic cells in the Arabidopsis root [2,32]. The identity of this gene remains unknown, but cytokinin has recently been shown to determine root meristem size by controlling cell differentiation [33]. On the contrary, our data suggest that GA controls root meristem size in rice through its dose-dependent effects on cell elongation and proliferation in the root meristem, which are mediated by SHB in a developmental stage-specific manner.

Recent studies in the Arabidopsis root have shown that GA controls root meristem size by modulating cell proliferation [3,14]. GA deficiency, either in GA biosynthesis mutants or induced by PAC treatment, significantly impairs cell proliferation in the Arabidopsis root, leading to a decrease in cell production rate and meristem size. Expression of a non-GA-degradable DELLA mutant in endodermal cells was sufficient to inhibit cell proliferation and block root meristem enlargement, indicating that GA controls root meristem size in a DELLA-dependent manner. A reduction in cell elongation in the root meristem was hypothesized to cause reduced number of cell division events and thus block the increase in meristem size [3]. However, several evidences from our study suggest that in rice GA could regulate cell elongation in the root meristem independently of its effect on cell proliferation to influence meristem growth: 1) *shb* had reduced levels of GA_3_ and GA_4_ in the root (Fig 2J). Exogenous application of GA_3_ to the *shb* root could restore the length of meristematic cortical cells and the size of root meristem to WT (Fig 2H and 2I). 2) Treating the WT root with 1 μM PAC could phenocopy the effect of the *shb* mutation, resulting in a shorter root meristem with reduced cortical cell length but unaltered cell number (Fig 6). 3) Higher concentrations of PAC (10–50 μM) significantly impaired cell proliferation in both WT and *shb* root meristems (Fig 6). It is also interesting to note that while Arabidopsis root meristem size nearly doubles during the first 4 days after sowing [34], rice root meristem size shows only a 10–20% increase over the same period (S2D Fig). The increase in Arabidopsis root meristem size was attributed to a proportional increase in meristematic cortical cell number [34], whereas the size of rice root meristem (S2D Fig) appears to be influenced by both the number (S2F Fig) and the SHB-regulated length (S2E Fig) of meristematic cortical cells.

How does SHB regulate GA levels and consequently cell elongation and proliferation in the root meristem? Our *in vitro* and *in vivo* data suggested that SHB directly binds to and activates transcription of the GA biosynthesis gene *KS1*, which has overlapping expression domain with *SHB* in the root meristem (Figs 4 and 5). In addition, our qPCR analysis indicated that SHB could also act through the GA biosynthetic gene *GA20OX2*/*SD1* to modulate GA production in the root meristem (Fig 4C), but the underlying molecular mechanism remains to be elucidated. Intriguingly, our qPCR analysis showed that both *SHB* and *KS1* expression were induced by GA but repressed by SLR1, the only DELLA protein in rice (Fig 7D). These findings suggest that GA regulates its own biosynthesis and consequently cell elongation and proliferation in the rice root meristem via positive feedback regulation on the expression of genes involved in the early steps of GA biosynthesis.

Taken together, our data suggest a model (Fig 7E) in which the root meristem size is modulated through dose-dependent effects of GA on cell elongation and proliferation in the root meristem, which are mediated by a developmental and possibly cumulative process that involves the root meristem- and developmental stage-specific function of the AP2/ERF transcription factor SHB. SHB, whose transcription is induced by GA, promotes GA biosynthesis in the root meristem by directly binding to and activating the GA biosynthesis gene *KS1*. SHB-dependent and KS1-mediated GA availability is well correlated with the ability of meristematic cortical cells to elongate beyond the minimal cell length requirement for normal cell proliferation, allowing optimal rice root meristem growth. Loss of *SHB* function at the early stage of meristem development reduces the root meristem size by reducing elongation of meristematic cortical cells, without causing apparent defect in cell proliferation. This phenotype can be mimicked in WT roots by a moderate reduction of endogenous GAs in the presence of 1 μM PAC. Loss of *SHB* function at a later stage of meristem development (from around the fifth day after sowing) impairs both cell elongation and proliferation and consequently, further reducing the root meristem size. The reduction of cortical cell number in the *shb* root meristem could be correlated to the increase of cortical cell length, indicating cell cycle arrest. Loss of *KS1* function and exposure of WT roots to high concentrations of PAC (10 or 50 μM), which strongly reduce endogenous levels of GAs, result in a more severe reduction of cortical cell number and a further elongation of cortical cells, suggesting that the degree of cell cycle arrest is related to the endogenous levels of GAs. The lower the GA levels, the more severe the cell elongation and proliferation defects and consequently, the smaller the root meristem size.

Because of the importance of plants on the global level to food security and environmental sustainability, exploring molecular mechanisms underlying the control of plant organ size and growth has become a high priority in plant research worldwide [35]. Given that most agriculturally important crop species are monocots, our finding that SHB and its putative orthologs in monocot crop species are closely clustered in phylogenetic tree is of great importance. Future studies on their functions may lead to the identification of evolutionarily conserved mechanisms in cell size control and further our understanding on how meristem growth is modulated without markedly compromising cell proliferation to influence organ and body size. Consequently, rational design of crop plant architecture may be enabled by modulating the activities of SHB and its orthologs, which may improve the ability of crop plants to cope with adverse weather conditions such as rain, wind and hail [36], ultimately leading to a second GA-dependent ‘Green Revolution’ in crop productivity.

## Materials and Methods

### Plant Materials and Growth Conditions

The mutant line 03Z11ER89 (*shb*) was isolated from a rice enhancer trap collection [37]. The *ks1*, *gid1* and *slr1* mutants were reported previously [19,29,30]. *ks1* mutants set no seeds and therefore the mutation were maintained in a heterozygous state. For field studies, rice plants were cultivated under natural long-day conditions during rice cultivation seasons at the experimental field of Huazhong Agricultural University. For the analysis of seedling root phenotypes, seeds were sterilized and sowed simultaneously (except for S2D–S2K Fig experiments which were conducted by sowing *shb* seeds 12 h earlier before WT controls) on petri plates containing half-strength Murashige and Skoog (MS) medium (Duchefa, The Netherlands), supplemented with 1% sucrose, 0.05% MES and 0.8% agar and adjusted to pH 5.8. The plates were then placed vertically and incubated at 28°C in either continuous darkness or a light/dark (14/10 h) regime, depending on the experimental design.

### Plasmid Construction and Rice Transformation

For the complementation of *shb* phenotypes, the genomic sequence of *SHB* gene, together with 2.51 kb promoter and 386 bp 3’-UTR regions, was inserted into the pCAMBIA2301 vector (http://www.cambia.org) to generate pCAMBIA2301-SHB. To construct the complementing SHB-GFP fusion, sunlight GFP was fused in-frame to the C terminus of *SHB* coding sequence, and subcloned into pCAMBIA2301 under the control of the 2.51 kb *SHB* promoter. The empty vector pCAMBIA2301 was used as a negative control. Transgenic rice plants carrying each of these constructs were produced by using *Agrobacterium*-mediated transformation of callus of *shb* mutant. To construct CPMV::SHB-GFP, the coding sequence of SHB was fused in-frame to the N terminus of GFP and subcloned into the pEAQ-HT-DEST1 vector by using the GATEWAY recombination system, under the control of the Cowpea Mosaic Virus (CPMV) promoter [25]. Primers used for the construction of these vectors are listed in S2 Table.

### Sequence and Phylogenetic Analyses

Protein sequences of putative orthologs of SHB from the other plant species were obtained by using blast search against the NCBI database (http://www.ncbi.nlm.nih.gov). Multiple protein sequence alignment was performed using the ClustalX Version 2.0 [38]. The alignment was then manually refined. A phylogenetic tree was constructed using the MEGA 4.0 program [39] with the following parameters: Poisson correction, pairwise deletion, and bootstrap (1000 replicates; random seed).

### Quantitative Analysis of Root Phenotypes

Root length was measured with Image J software (http://rsb.info.nih.gov/ij). Root meristem size was determined by measuring the length from the quiescent center to the first elongated epidermal cell. Cell number in the root meristem, average cell length and width in the root meristem were quantified with all cells in the fourth cortical layer of the root meristem. Cell production rate in the rice root was calculated as described previously [3,40]. Briefly, time-course analyses of root growth and mature cell length were performed and root growth rate was determined using a five-point equation. Cell production rate was then calculated using the following equation: cell production rate = root growth rate/ mature cell length. Cells in the fourth cortical layer of the root elongation zone and maturation zone were used to obtain the quantification data of average cell length in the root elongation zone and mature cell length, respectively. For each quantification, at least 15 rice plants were analyzed.

### 5-ethynyl-2′-deoxyuridine (EdU) Staining

EdU staining was performed using an EdU kit (C10310, Apollo 488) from Ribobio, China, according to the manufacturer’s protocol. Briefly, roots of 4-day-old rice seedlings were immersed in 50 μM EdU solution for either 2 h (Figs 1F, 1G and 5G and 5H) or 20 h (Fig 6C and 6D), and then fixed for 30 min in 4% paraformaldehyde, followed by 30 min of incubation with Apollo. The samples were next hand-sectioned longitudinally and EdU images of the sections were then captured with a Leica TCS SP2 confocal laser-scanning microscope equipped with a 20× water immersion objective and analyzed with Leica LAS AF software. Quantification of numbers of EdU-stained cells was performed in the fourth cortical cell layer of the rice root meristem, in a selected portion with a length of 360 μm.

### Quantification of Endogenous GAs

Quantification of GAs in the WT, *shb* and *ks1* was performed as described previously [41], using [^2^H_2_] GA_1_ (1.00 ng/g), [^2^H_2_] GA_3_ (1.00 ng/g), [^2^H_2_] GA_4_ (1.00 ng/g) [^2^H_2_] GA_12_ (2.00 ng/g), [^2^H_2_] GA_24_ (2.00 ng/g), [^2^H_2_] GA_19_ (5ng/g), [^2^H_2_]GA_20_ (2ng/g), [^2^H_2_]GA_34_ (2ng/g), [^2^H_2_]GA_44_ (2ng/g) and [^2^H_2_] GA_53_ (2.00 ng/g) as internal standards. Roots of 7-day-old seedlings were used for comparison between the WT and *shb*. To compare the levels of endogenous GAs in *ks1*, *shb* and the WT, whole seedlings were used due to the severity of *ks1* root phenotype.

### Chemical Treatment

Rice seeds were sown on medium supplemented with 10 μM GA_3_ and cultured for 4 days before analyzing the effect of GA on the root phenotypes. To determine the minimum concentration of GA required to rescue *shb* and *ks1* root phenotypes, 3-day-old seedlings were cultured on medium supplemented with 10, 25, 50, 75 or 100 μM GA_3_ for 24 hours before analysis. To examine the dose-effects of PAC on rice roots, 3-day-old seedlings were cultured on medium supplemented with 0.1, 1, 10 or 50 μM PAC for 24 hours before analysis. For mock treatments, medium with ethanol at the final concentration as for chemical treatments was used.

### Quantitative Real-Time PCR (qPCR)

Total RNA was extracted from WT and mutant roots using TRIzol (Invitrogen) reagent, according to the manufacturer’s instructions. qPCR analysis was performed in a 96-well plate with an ABI StepOnePlus Real-Time PCR System (Applied Biosystems). The following thermal profile was used for all reactions: 95°C for 10 min, 40 cycles of 95°C for 15 s and 60°C for 1 min. The melting curve was determined under the following conditions: 95°C for 15 s, 60°C for 1 min, and 95°C for 15 s. The rice *ubiquitin1* gene (*Os03g13170*) was used as the internal control. All primers used are listed in S2 Table.

### RNA *In Situ* Hybridization

RNA *in situ* Hybridization was performed as described previously [42]. Briefly, roots of 4-day-old rice seedlings were fixed in FAA (50% ethanol, 5% acetic acid and 3.7% formaldehyde) at 4°C for 24 h, dehydrated in an ethanol series, cleared through a xylene series and then embedded in paraffin. 8 to 12 μm sections were mounted on RNase-free glass slides and *in situ* hybridization was then performed using digoxigenin-labeled RNA probes transcribed with either T7 or SP6 transcriptase from pGEM-T plasmids containing part of the *SHB* or *KS1* coding sequence, which were PCR amplified with gene-specific oligonucleotide pairs SHBinsitu-F/R and KS1insitu-F/R (S2 Table).

### Subcellular Localization Analysis

Subcellular localization analysis of SHB-GFP was performed as previously described [43]. Briefly, lower leaves of *N*. *tabacum*. plants were infiltrated with *Agrobacterium* strains carrying CPMV-SHB-GFP using a syringe. For co-localization analysis with the nuclear marker RFP-SRT1 [26] the bacteria were mixed in appropriate volumes of infiltration buffer prior to injection into the leaves. Expression of fluorescent proteins was captured 2–3 d after infiltration with a Leica TCS SP2 confocal laser-scanning microscope equipped with a 40× water immersion objective and processed with Leica LAS AF software.

### Electrophoretic Mobility Shift Assay (EMSA)

To detect the binding of SHB protein to the *KS1* promoter, EMSA was performed as described previously [42] with recombinant SHB protein produced in *E*. *coli* DE3 cells (Novagen). In brief, the recombinant SHB protein was incubated with an [α ^32^P]-radiolabeled, double-stranded DNA oligonucleotide that covers the region containing the putative SHB binding sequence (GCCGCC) in the *KS1* promoter. For control EMSA, nucleotide substitutions were introduced into the putative SHB binding site to produce the control probe. DNA binding reactions were carried out at room temperature for 20 min and the separation of protein-DNA complexes from the free DNA probes was done by non-denaturing polyacrylamide gel electrophoresis followed by auto-radiographic detection.

### Chromatin Immunoprecipitation (ChIP)-qPCR

Chromatin extraction and immunoprecipitation were performed as previously described [26]. Briefly, roots of the SHB-GFP plants were first vacuum-infiltrated and fixed in formaldehyde. The chromatin was then isolated from the nuclei of root cells and pre-cleared with sheared salmon sperm DNA/protein A agarose (Invitrogen), and immunoprecipitated with or without an anti-GFP antibody (Abcam; ab290). The protein/DNA complexes were eluted and crosslinks were reversed to free DNA. The immunoprecipitated DNA was then purified and qPCR was performed with primers against *KS1* promoter region. qPCR was also performed with input DNA purified from the pre-cleared chromatin, and the rice *Actin* gene (*Os11g06390*) was used as the reference gene for normalization of qPCR data.

### Western Blot Analysis

Rice root nuclear proteins were extracted from roots of 7-day-old WT and SHB-GFP transgenic plants. Western blot analysis was performed as described previously [26]. After washing in acetone and dried, the proteins were resuspended in Laemmli sample buffer, then separated on a 12% SDS-PAGE and transferred to an Immobilon-P PVDF transfer membrane (Millipore). The membrane was blocked with 2% bovine serum albumin in phosphate-buffered saline (pH 7.5), and incubated overnight with primary antibodies, such as anti-GFP (Abcam; ab290), in a 1:5,000 dilution at room temperature. After three washes (30 min each), the secondary antibody (goat anti-rabbit IgG [SouthernBiotech]) at 1:10,000 dilution was used. Visualization was performed using the Super Signal West Pico kit (Pierce) according to the manufacturer’s instructions.

## Supporting Information

S1 Fig The *shb* mutant has characteristics of rice GA-deficient or insensitive mutants. (A) Phenotype of aerial parts of WT and *shb* plants at maturity. The *shb* mutant has a dwarf phenotype. Scale bar = 15 cm. (B) Comparison of internode lengths between WT and *shb*. From left to right, the uppermost, second, third and fourth internodes of WT and *shb*. All internodes from the *shb* mutant are shorter than corresponding WT controls. Scale bar = 5 cm.
(TIF)

S2 Fig The *shb* mutant showed delayed seed germination and development stage-dependent root phenotypes. (A-C) Comparison of seed germination in WT and *shb* mutants. Note that *shb* germinated approximately 12 h later than the WT. Scale bar = 1 cm. (D-K) Time-course analysis of root meristem size (D), meristematic cortical cell length (E) and number (F), root length (G), root growth rate (H), cell production rate (I) and cortical cell length in the root elongation zone (J) and maturation zone (K) of WT and *shb* seedlings following synchronized seed germination. Bars with different letters are significantly different at *P* < 0.05, *t*-test.
(TIF)

S3 Fig SHB and its putative orthologs in monocot crop species are closely clustered in phylogenetic tree. (A) Multiple sequence alignment of SHB and its putative orthologs from date palm (Genebank gi: 672165458), banana (695078910), wild rice (573943989), maize (226510301), sorghum (242087803), foxtail millet (514750833), barley (326509149), tausch’s goatgrass (475559664), purple false brome (357129306) and Arabidopsis (21593696). Red dashed line indicates the AP2 domain. (B) Phylogenetic analysis of SHB and its putative orthologs from other plant species. Bootstrap values are indicated on branches. The scale bar of 0.05 is equal to 5% sequence divergence.
(TIF)

S4 Fig Expression of the SHB-GFP fusion protein functionally complemented the *shb* mutant phenotypes. (A) Western blot analysis of the expression of the SHB-GFP fusion protein (under the control of the native *SHB* promoter) in roots of the WT and SHB-GFP plants. (B) Root meristem size in 4-day-old WT, SHB-GFP and *shb* seedlings. C) Average cell length in the root meristem of 4-day-old WT, SHB-GFP and *shb* seedlings. Bars with different letters are significantly different at *P* < 0.05, *t*-test.
(TIF)

S5 Fig *KS1* had reduced expression level and domain in the *shb* root. (A) RNA *in situ* hybridizations in the WT root with a *ks1* anti-sense probe. (B) RNA *in situ* hybridizations in the *shb* root with a *ks1* anti-sense probe. (C) RNA *in situ* hybridizations in the WT root with a *ks1* sense probe. Scale bar = 100 μm.
(TIF)

S6 Fig KO2 was strongly up-regulated in *ks1* roots as compared to the WT control. qPCR analysis of transcript levels of GA biosynthetic genes *KO2* in 4-day-old WT and *ks1* roots. Transcript levels from the WT were set to 1. Error bars represent SD from three independent experiments. **, *P* < 0.01, *t*-test.
(TIF)

S7 Fig A higher concentration of GA_3_ was needed to restore the root meristem size in *ks1* than in *shb*. (A) Root meristem size of 3-day-old WT and *ks1* seedlings treated with mock or GA at indicated concentrations for 24 hours. (B) Root meristem size of 3-day-old WT and *shb* seedlings treated with mock or GA at indicated concentrations for 24 hours. Bars with different letters are significantly different at *P* < 0.05, *t*-test.
(TIF)

S8 Fig PAC-induced root phenotypes could be reversed by co-treatment with GA. (A) Root meristem size of 3-day-old WT seedlings treated with mock or co-treated with PAC and GA at indicated concentrations for 24 hours. (B) Cell number in the root meristem of 3-day-old WT seedlings treated with mock or co-treated with PAC and GA at indicated concentrations for 24 hours. (C) Average cell length in the root meristem of 3-day-old WT seedlings treated with mock or co-treated with PAC and GA at indicated concentrations for 24 hours. Bars with different letters are significantly different at *P* < 0.05, *t*-test.
(TIF)

S1 Table Levels of endogenous GAs in 7-day-old WT, *shb* and *ks1* seedlings. (TIF)

S2 Table Primers and probes used in this study. (TIF)
