# Supplementary figures and images for: SHOEBOX Modulates Root Meristem Size in Rice through Dose-Dependent Effects of Gibberellins on Cell Elongation and Proliferation
Source: PLoS Genet. 2015 Aug 14;11(8):e1005464. doi: 10.1371/journal.pgen.1005464 (PMC4537253; doi:10.1371/journal.pgen.1005464)

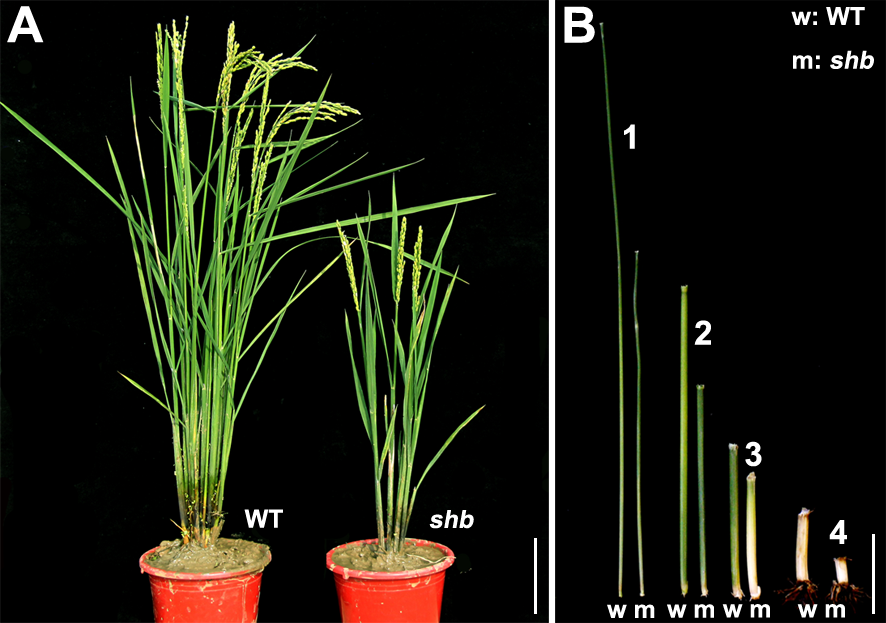

Supplement: S1 Fig — (A) Phenotype of aerial parts of WT and shb plants at maturity. The shb mutant has a dwarf phenotype. Scale bar = 15 cm. (B) Comparison of internode lengths between WT and shb. From left to right, the uppermost, second, third and fourth internodes of WT and shb. All internodes from the shb mutant are shorter than corresponding WT controls. Scale bar = 5 cm. (TIF) [file pgen.1005464.s001.tif]

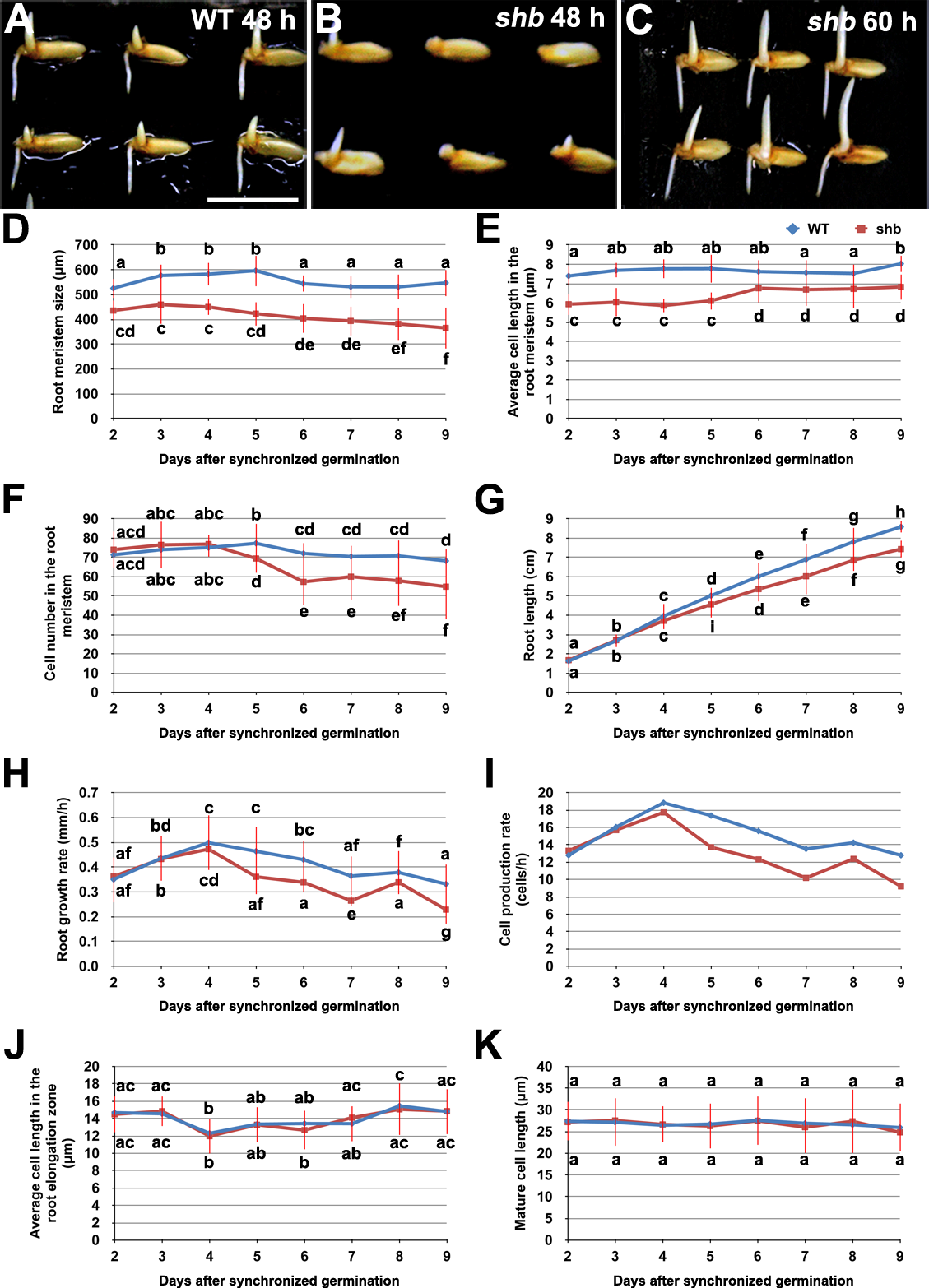

Supplement: S2 Fig — (A-C) Comparison of seed germination in WT and shb mutants. Note that shb germinated approximately 12 h later than the WT. Scale bar = 1 cm. (D-K) Time-course analysis of root meristem size (D), meristematic cortical cell length (E) and number (F), root length (G), root growth rate (H), cell production rate (I) and cortical cell length in the root elongation zone (J) and maturation zone (K) of WT and shb seedlings following synchronized seed germination. Bars with different letters are significantly different at P < 0.05, t-test. (TIF) [file pgen.1005464.s002.tif]

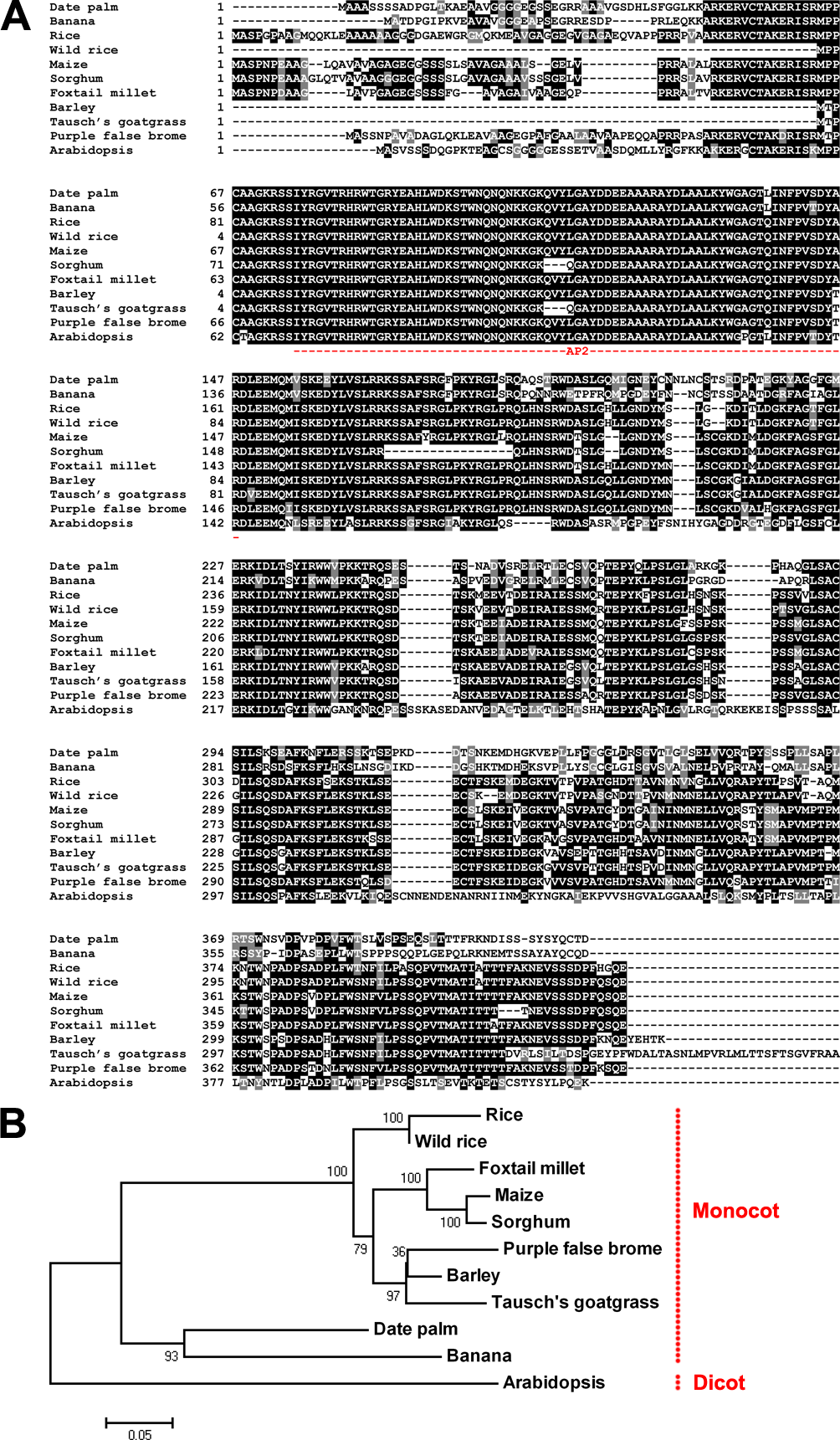

Supplement: S3 Fig — (A) Multiple sequence alignment of SHB and its putative orthologs from date palm (Genebank gi: 672165458), banana (695078910), wild rice (573943989), maize (226510301), sorghum (242087803), foxtail millet (514750833), barley (326509149), tausch’s goatgrass (475559664), purple false brome (357129306) and Arabidopsis (21593696). Red dashed line indicates the AP2 domain. (B) Phylogenetic analysis of SHB and its putative orthologs from other plant species. Bootstrap values are indicated on branches. The scale bar of 0.05 is equal to 5% sequence divergence. (TIF) [file pgen.1005464.s003.tif]

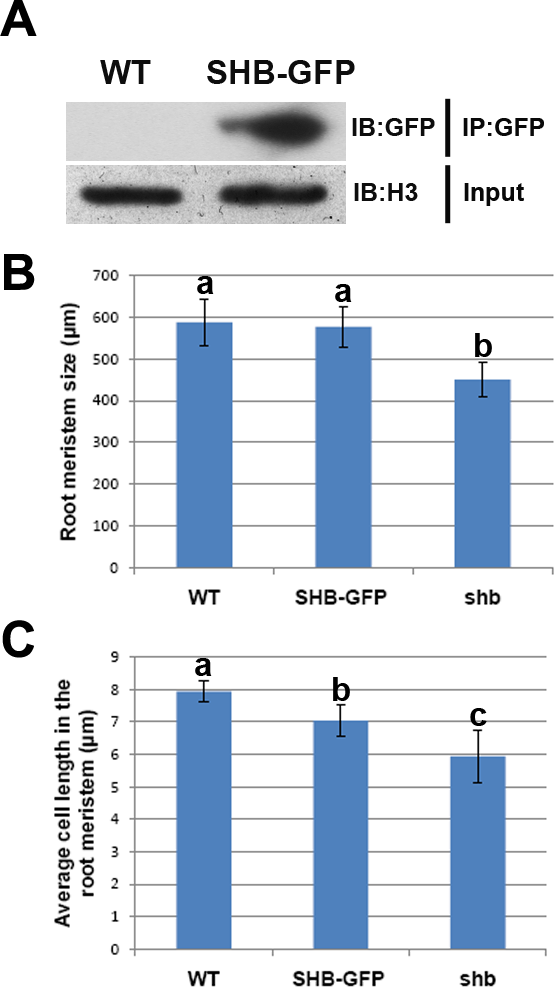

Supplement: S4 Fig — (A) Western blot analysis of the expression of the SHB-GFP fusion protein (under the control of the native SHB promoter) in roots of the WT and SHB-GFP plants. (B) Root meristem size in 4-day-old WT, SHB-GFP and shb seedlings. C) Average cell length in the root meristem of 4-day-old WT, SHB-GFP and shb seedlings. Bars with different letters are significantly different at P < 0.05, t-test. (TIF) [file pgen.1005464.s004.tif]

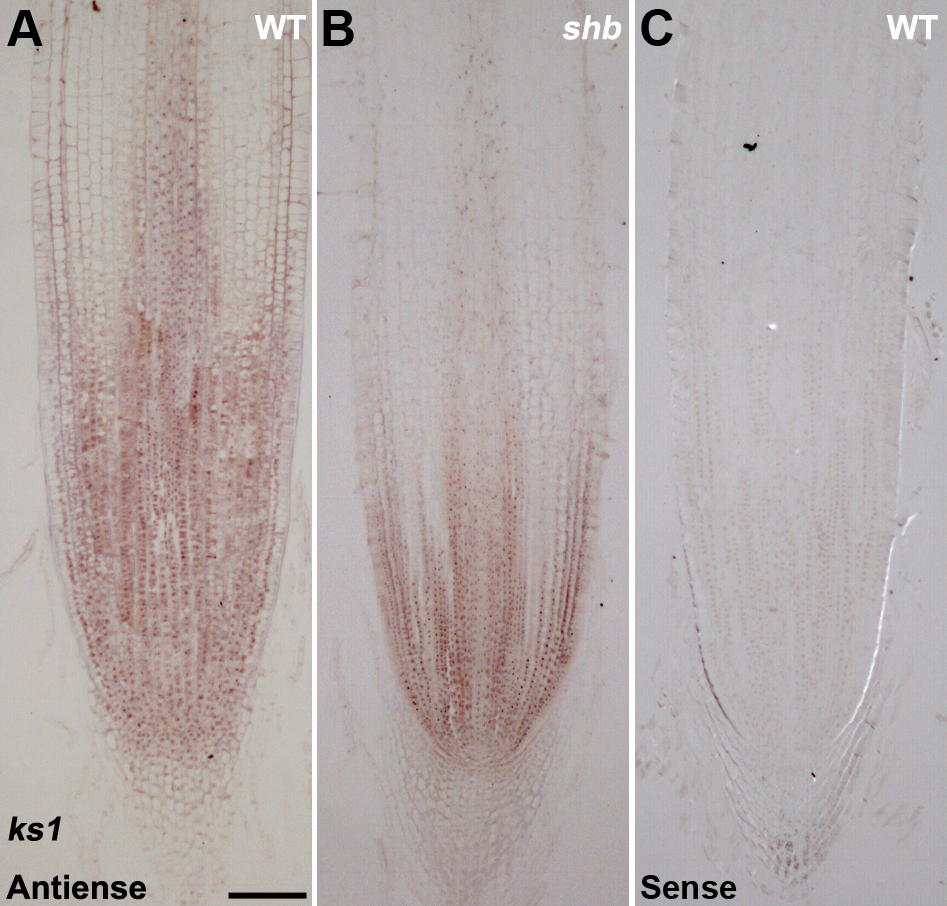

Supplement: S5 Fig — (A) RNA in situ hybridizations in the WT root with a ks1 anti-sense probe. (B) RNA in situ hybridizations in the shb root with a ks1 anti-sense probe. (C) RNA in situ hybridizations in the WT root with a ks1 sense probe. Scale bar = 100 μm. (TIF) [file pgen.1005464.s005.tif]

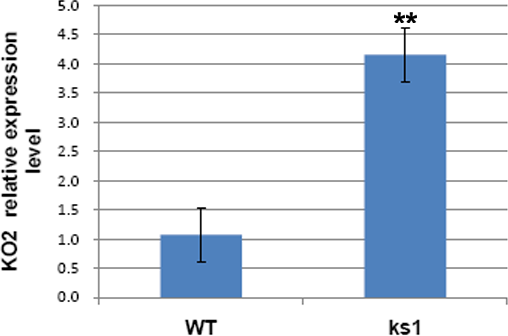

Supplement: S6 Fig — qPCR analysis of transcript levels of GA biosynthetic genes KO2 in 4-day-old WT and ks1 roots. Transcript levels from the WT were set to 1. Error bars represent SD from three independent experiments. **, P < 0.01, t-test. (TIF) [file pgen.1005464.s006.tif]

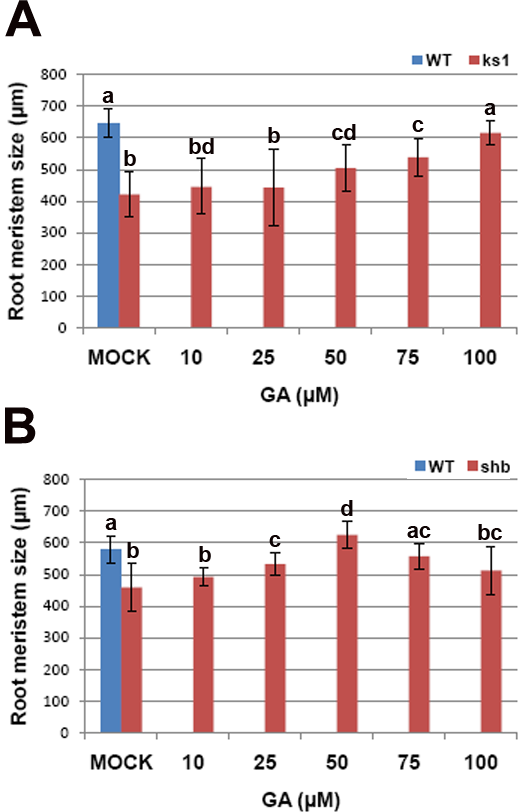

Supplement: S7 Fig — (A) Root meristem size of 3-day-old WT and ks1 seedlings treated with mock or GA at indicated concentrations for 24 hours. (B) Root meristem size of 3-day-old WT and shb seedlings treated with mock or GA at indicated concentrations for 24 hours. Bars with different letters are significantly different at P < 0.05, t-test. (TIF) [file pgen.1005464.s007.tif]

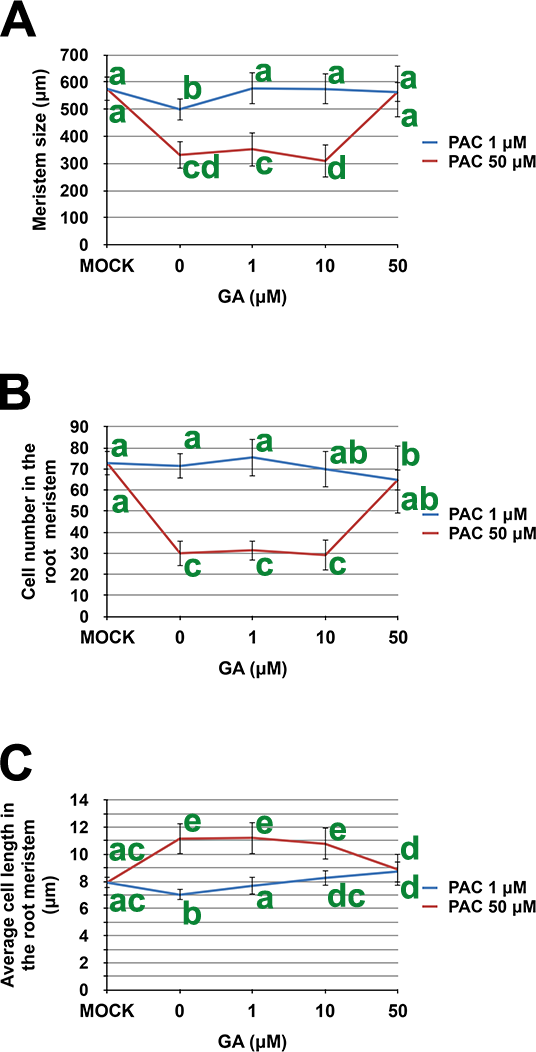

Supplement: S8 Fig — (A) Root meristem size of 3-day-old WT seedlings treated with mock or co-treated with PAC and GA at indicated concentrations for 24 hours. (B) Cell number in the root meristem of 3-day-old WT seedlings treated with mock or co-treated with PAC and GA at indicated concentrations for 24 hours. (C) Average cell length in the root meristem of 3-day-old WT seedlings treated with mock or co-treated with PAC and GA at indicated concentrations for 24 hours. Bars with different letters are significantly different at P < 0.05, t-test. (TIF) [file pgen.1005464.s008.tif]

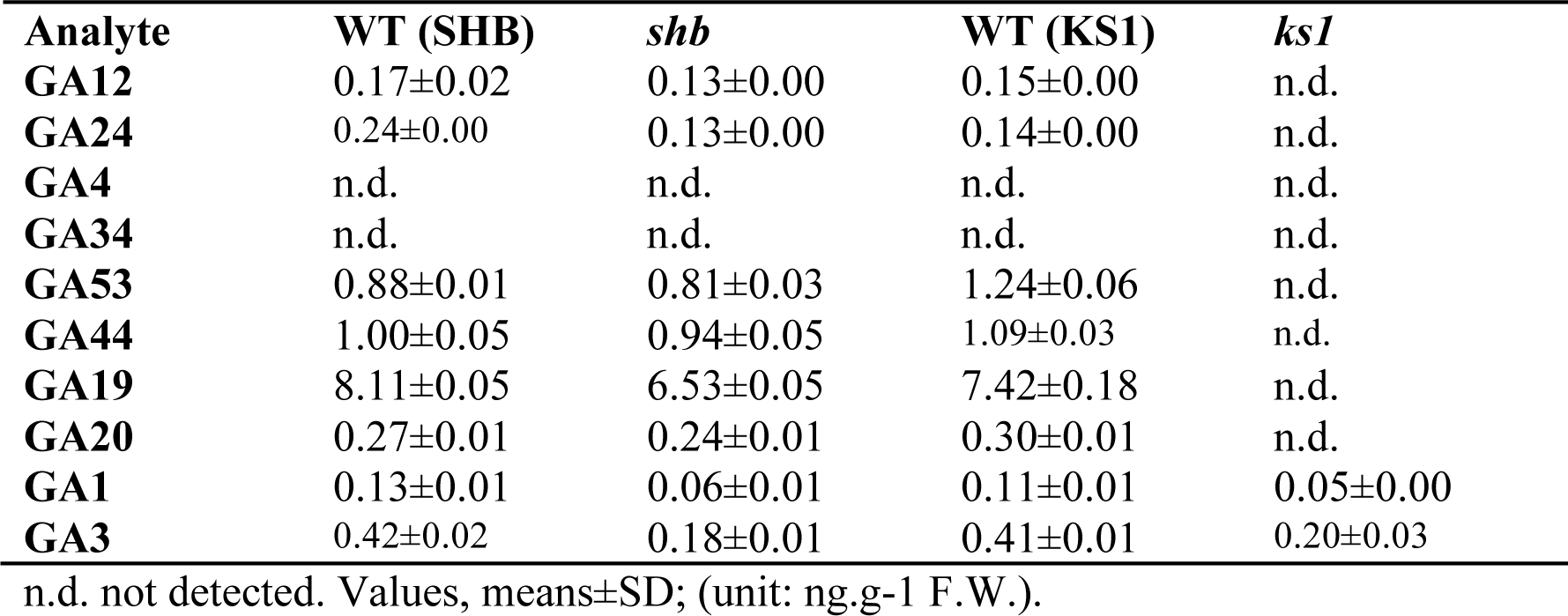

Supplement: S1 Table — (TIF) [file pgen.1005464.s009.tif]

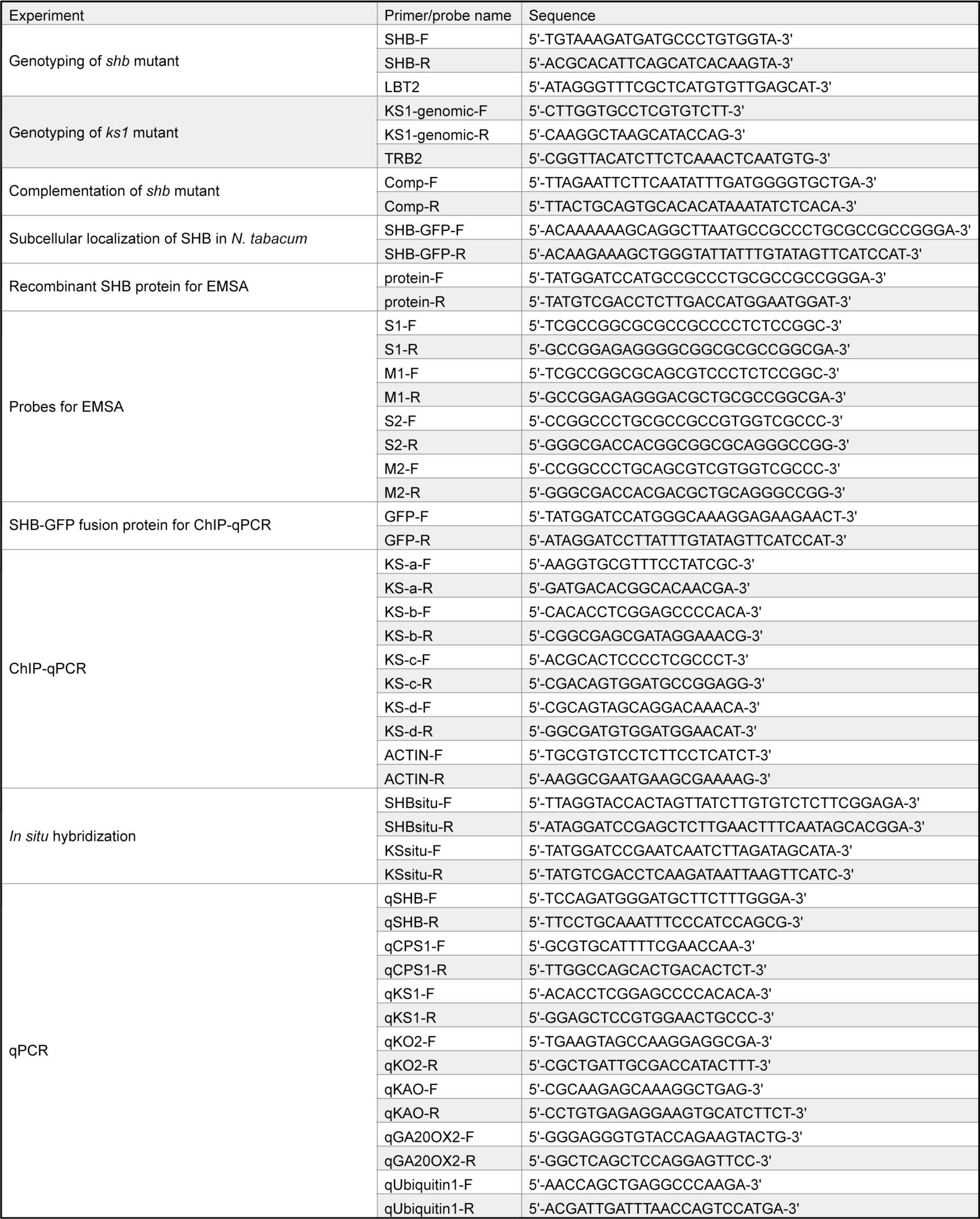

Supplement: S2 Table — (TIF) [file pgen.1005464.s010.tif]
